# The relationship between psycho-environmental characteristics and wellbeing in non-spending players of certain mobile games

**DOI:** 10.1098/rsos.221129

**Published:** 2023-01-25

**Authors:** Elena Petrovskaya, David Zendle

**Affiliations:** University of York, York, UK

**Keywords:** microtransactions, video games, playtime, media effects

## Abstract

Revenue generation in modern digital games is often dependent on in-game continuous player spending. This brings concerns that games may be including features which drive player spending in potentially harmful ways. Moreover, it is unknown what types of individuals may be vulnerable to these design-driven harms. We used player-donated, objective data of playtime and in-game spending from a sample of 295 players of games previously identified as ‘designed to drive spending’. We combined this with psycho-environmental characteristics and wellbeing measures administered to the players. Quantile regression analyses did not show an interaction between player characteristics and playtime/spend as predictors of wellbeing outcomes; nor did we find a difference in wellbeing between players of these games and games with alternative monetization models. This is discussed in light of a low proportion of spenders in the sample, which may affect results pertaining to the moderating role of spend. However, it suggests that while design features in games aiming to drive player spend may be unethical and problematic, they may not necessarily cause harm to the normative player.

## 1. Introduction

Contemporary video games are often monetized by continuous, repeated in-game purchases rather than flat fees for the purchase of the game itself. This creates reliance on in-game purchases—*microtransactions*—as a source of revenue generation. Logically, it leads to developers focusing on making in-game spending a desirable option for players, and one which they are likely to engage with often.

This means that on the industry side of the production of these games, player activity becomes value generation, and consequently becomes used as a mechanism for control over player behaviour [1]. As free-to-play games translate player effort, personal information and needs into revenue streams, games are being designed to increase time-on-device, player retention and conversion from free to paid play [2]. Technological developments have also made it easier to incorporate gambling-like elements into digital games, primarily through in-app purchases of items like skins and in-game currency which can be used for gambling simulation [3]. These shifting design incentives and technological advances are contributing to the ‘gamblification’ of digital games: a growing overlap between gaming and gambling mechanics and practices [4].

So, designing games to encourage player investment has become a greater priority. However, there have been some concerns regarding the effects of such design, and how far might be *too far* in driving player spending. Previous work has highlighted that many microtransactions are perceived negatively by players, for reasons such as being unfair, aggressive or misleading [5]; and indeed that 52% of mobile games are seen as having their ‘game dynamics designed to drive spending’: game environments which players believe are designed to facilitate player spend, rather than for the improvement of their gameplay experience [6]. There have also been direct links found between engagement with certain types of microtransactions, such as loot boxes (in-game items bought with real money containing randomized contents) and token wagering (wagering in-game tokens which can then be redeemed for in-game rewards), and problem gambling [7]. These trends point to a need for a deeper investigation of player wellbeing after interaction with game mechanics designed to encourage player investment of time and money.

### 1.1. Playtime, money and wellbeing

The current study builds on our previous work [8], which consisted of interviews with players of games that had had ‘their dynamics designed to drive spending’. This work resulted in a grounded theory of five life areas of problematic consequences—financial, emotional, educational/vocational, sleep and social—which were a result of player interaction with such games and the monetization methods they employed. Interestingly, these problematic consequences were a result of not purely financial investment because of pressure by the game: some of the design elements in this category offer a choice to the player of whether they wish to obtain the same objective by spending a lot of time playing, or by spending money. These include being forced to put an ‘unpleasantly large amount of time and effort into completing a portion of [a] game’, or optionally completing a transaction to avoid this unpleasant ‘grind’; and ‘given the choice of waiting some time before being able to progress in game or paying some money to skip this wait’ [5]. As such, some of the problems to wellbeing occur because of excessive time invested into play—but this time investment is also a result of design to drive spending.

The present work seeks to further investigate how the above-described wellbeing outcomes are related to time and financial investment into the games. In particular, we are interested in testing whether certain types of people would be *particularly* vulnerable to the design elements used in games to drive player spending.

Our work joins a larger academic discourse on whether playtime has an effect on player wellbeing. A recently published study by Vuorre *et al*. [9], which tracked 38 935 players across seven games for six weeks in playtime and linked it to affective wellbeing and general life satisfaction, found that time spent playing had ‘limited to no impact on wellbeing’. The authors conclude ‘limiting or promoting play based on time alone appears to bear neither benefit nor harm’. However, this is in discord with work into gaming disorder, which is associated with heavy gameplay (e.g. [10]), and also with various problems with wellbeing, like health consequences [11], problems with academic and career achievements [12], and psychosocial problems [13]. This is probably due to a series of interconnected factors, rather than a direct link between heavy gaming and consequences for wellbeing. Nonetheless, such a body of research is too compelling to ignore, and identifying what these factors are should therefore be a priority.

The current work therefore aims to contribute to this goal by considering the role of the *game*: could the medium be what causes the distinction between healthy heavy gaming and excessive, disordered play?

### 1.2. Psycho-environmental characteristics and vulnerability

Another piece in the puzzle might be the psychological and environmental characteristics of the people playing the games. In our previous work [8], we found that in playing games which had elements designed to drive player spend, only certain people were vulnerable to being affected by these elements, while others were able to engage and disengage as they wanted. These characteristics consisted of mental health problems, stress at work, low self-esteem and poor quality of life. Other contributing factors included being female—nearly all of the affected participants identified as female, and not having children that the participant regularly saw.

This is in line with work into gaming disorder, which consistently suggests that not all individuals who play games will experience gaming-related problems, but a small subset will. Those include players experiencing mental health problems, lower life satisfaction and stress [14,15].

However, the current evidence base does not provide a comprehensive understanding of which psycho-environmental characteristics correlate particularly with being affected by *design elements* of games which encourage spending. This information could be helpful to policy makers and regulators in implementing evidence-driven interventions. A more granular understanding of this could also protect games in general from being pathologized (a common concern for critics of gaming disorder [16], while ensuring the populations who need protection are protected. A model for such an approach can be followed from gambling, where the role of the design of gambling machines in keeping players invested is well-acknowledged [17]. It is a robust finding that disordered gambling is more prevalent across certain groups [18], which has subsequently been used in arguments for ‘interventions to assist at-risk and problem gamblers' [19].

### 1.3. A need for objectivity

Furthermore, there is a need for *objectivity* in the measurement of playtime and spending. A common issue in the study of gaming and wellbeing is the reliance on self-report measures, which are notoriously inaccurate for this purpose [20]. This issue spans across all technology use [21], but research in time spent using other types of media has adapted to use innovative methods for an accurate assessment.

One example of such a method is the use of built-in screen-time functions in mobile devices. Many phone operating systems offer either built-in or downloadable apps which allow tracking of how much time is spent on a specific app, which makes it useful for tracking either screen time spent on certain activities, or in general. For example, Ohme *et al*. [22] use this method to assess user accuracy in reporting smartphone usage, relying on user ‘data donation’.

This method can be generalized to games, and in the case of the current work comes in useful, as we were interested specifically in mobile games. Because of this approach, we were able to gather objective data of (i) player time on certain games, and (ii) player spend. Both of those types of data are difficult to gather in studying games (particularly spend), as companies are often reluctant to share this data because it is high risk and low reward for them [23]. As such, although working with large amounts of industry data would doubtlessly lead to high-quality research, it is currently only available to researchers in privileged positions. Using player-donated objective data provides a workaround to this problem and overcomes the problem of subjectivity and inaccuracy.

### 1.4. The current study

We conducted a pre-registered survey of 295 players of games which had been identified by Petrovskaya *et al*. [6] as having had their ‘dynamics designed to drive spending’. Such games were initially identified through bottom-up work with players who were asked what microtransactions they had encountered in games that were unfair, misleading, or aggressive [5]. In follow-up work analysing player reviews for discussion of such features, it was found that 52% of top-grossing mobile games are perceived by players as designed to drive spending [6]. These games include design elements such as energy timers and ‘pay or grind’ (the choice between playing what seems an unrealistic amount of paying to skip this).

We collected psycho-environmental characteristics as measures of vulnerability, demographic information and wellbeing, as well as objective measures of time and financial spend, obtained through participants providing screenshot evidence.

We were interested in using objective evidence of time and spending to understand two research questions. Firstly, we wanted to know how psycho-environmental factors interact with time and monetary investment into games ‘designed to drive spending’ to lead to consequences for different areas of wellbeing. We used the factors from our prior qualitative work: mental health problems, stress at work, low self-esteem and poor quality of life, gender and having children. We also added impulsivity as a factor: given the very well-established links of this factor to problem gambling, gaming disorder and loot boxes [14,23,24], we had reason to believe it might be important in this context also. Secondly, we were interested in whether players of games ‘designed to drive spending’ would experience greater problems with wellbeing than players of games which had not been characterized as such.

Contrary to our hypotheses, there was no evidence to suggest that players with certain psycho-environmental traits were more likely to experience lower wellbeing outcomes when playtime/spend was taken into account. We also did not find a significant difference in wellbeing between players of games ‘designed to drive spending’ and players of alternative games. Taken together, these findings suggest there was not a relationship in our sample between psycho-environmental characteristics and wellbeing—which goes against the findings in our prior work—meaning the relationship may be either minimal, or present in specific subpopulations different to the one we sampled from. We provide possible explanations for this result, including characteristics of our target population and the social context of the pandemic.

## 2. Methods

The study was pre-registered (https://osf.io/nkc86/). It was an online survey.

### 2.1. Participants

Participants were recruited from two sources: the online bulletin board Reddit, and the participant database Prolific (although ultimately, there was a much higher proportion of Prolific participants). Reddit participants were recruited specifically from subReddits of ‘games which are perceived by players as having been designed to drive spending’ (the full list of such games can be found at https://osf.io/z7gqe/).

One of the motivations for this sampling strategy was that we were interested in studying as representative a spread of adult players as possible, as much previous work has focused on dysregulated players or adolescents. A large proportion of the population play our games of interest, and we wanted to see if the nature of ‘dynamics designed to drive spending’ would affect a normative player who interacts with them.

A total of 295 players were included in the analysis for hypotheses 1–6 (from a total of 727 initial respondents). This number was based on a power analysis of five predictors, alpha level of 0.01 and an f squared of 0.0625 for a linear multiple regression and a proposed power level of 0.80. Of those respondents, 157 were female, and the age range was 18–60 (average = 27). Two hundred and twenty-five respondents did not have children that they regularly saw.

In addition, 72 players of other games were recruited for comparison of wellbeing (hypothesis 7): the rationale for this was a power analysis of a one-tailed *t*-test to detect effect size of 0.4, at alpha = 0.01 and power of 0.9. The games considered were non-mobile: desktop and console games. The rationale for this sample was taken from Petrovskaya *et al*. [6], who found that desktop games had a much lower prevalence of ‘dynamics designed to drive spending’: only 10%.

Readers can access a synthetic dataset for the main sample, the control sample and the code used for analysis at https://tinyurl.com/peerreviewconsequences (made using the ‘synthpop’ R package [25]). Synthetic datasets are datasets generated based on real datasets, and preserving their statistical properties and the relationships between variables, allowing researchers to replicate analyses when there is risk related to sharing real data [26]. In this case, the datasets were synthesized prior to being standardized, and the synthesized variables were then standardized and summed, as detailed below. We reran our analyses on the synthetic datasets and found very similar results. This, coupled with visual inspection of the variable distribution of the synthetic variables alongside the original, leaves us confident the synthetic datasets closely resemble the original data.

### 2.2. Measures

Participants completed a battery of measures in the survey.

Psycho-environmental characteristics which may increase vulnerability, used in hypotheses 1–4:

— Life satisfaction was measured with the Satisfaction with Life Scale [27]. This is a five-item scale designed to measure global cognitive judgements of one's life satisfaction. Agreement is rated on a 7-point scale that ranges from 7 (strongly agree) to 1 (strongly disagree).
— Self-esteem was measured with the Single-Item Self-Esteem Scale [28]. This is a one-item measure of global self-esteem. Although one-item, it has strong convergent validity with the Rosenberg Self-Esteem Scale (a 10-item scale) and similar predictive validity [28].
— Impulsivity was measured with the Barratt Impulsiveness Scale [29], the most widely cited instrument for the assessment of impulsiveness which consists of 30 items in total. The scale is made up of six first-order factors: attention, cognitive instability, motor, perseverance, cognitive complexity and self-control.
— General psychopathology was measured with the Depression Anxiety Stress Scale (DASS-21 version) [30]. It is a set of three self-report scales designed to measure the emotional states of depression, anxiety and stress.
— Job satisfaction was measured with the Brief Index of Affective Job Satisfaction [31], a four-item scale rated from 5 (strongly agree) to 1 (strongly disagree).

Wellbeing measures, used in hypotheses 5–7:

— Financial wellbeing was measured using the InCharge Financial Distress/Financial Well-Being Scale [32]), an eight-item scale where items are rated 1–10 with higher scores indicating greater financial wellbeing. The scale measures ‘a latent construct representing responses to one's financial state on a continuum ranging from overwhelming financial distress/lowest level of financial well-being to no financial distress/highest level of financial well-being’.
— Emotional wellbeing was measured using the Warwick-Edinburgh Mental Well-being Scale [33], a 14-item scale with 5 response categories, summed to provide a single score. The items cover both feeling and functioning aspects of mental wellbeing.
— Employment/education-related wellbeing was measured using the ‘organizational skills' and ‘efficiency’ subscales of the Job Performance Scale [34] (eight questions in total, rated from 7 ‘strongly agree’ to 1 ‘strongly disagree’). These particular subscales were selected as they were the ones deemed most relevant to the qualitative conceptualization of vocational wellbeing from previous work.
— Sleep wellbeing was measured using the Single-Item Sleep Quality Scale [35], a one-item sleep quality assessment ‘which possesses favourable measurement characteristics relative to lengthier sleep questionnaires'.

In general, we wanted to find a balance between using the most valid measures and maximizing player survey completion, hence why we opted for brief/single item measures where they showed similar validity levels to longer questionnaires.

**Figure 1.**
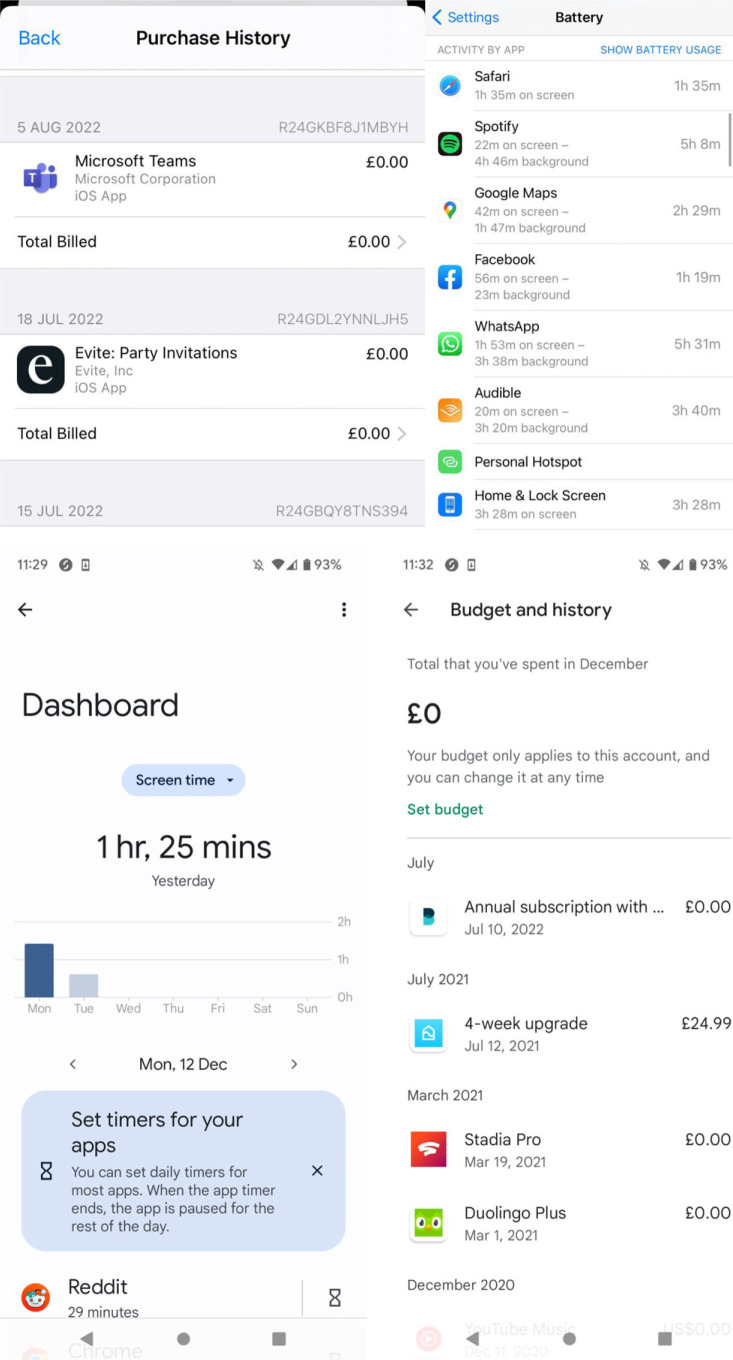
An example of the way a mobile phone might present the time and money a user had spent on a specific app (top two images are iPhone; bottom two are Android). Note: the screenshots are provided as an example by one of the authors, not from a participant.

A core aspect of our research was the objective measurement of time and money invested by players into the games. We asked participants to upload a screenshot of their activity and spend on the relevant game(s), as generated by their device: the majority of mobile phones allow users to see how much time they have spent on certain apps in a recent period, and can also track spend (figure 1).

Demographic information—age, gender, occupation and whether or not the participant had children which they regularly saw—was also collected.

### 2.3. Analysis

#### 2.3.1. Pre-processing

The data were processed prior to analysis. Respondents were removed on the basis of failing at least one attention check, or uploading the wrong screenshot evidence of their time playing and spending—for example, many participants did not convert their time in game from battery percentage to minutes before taking the screenshot.

The participant time and money spent on the game was also manually processed from the screenshots into an analysable format. The time invested was converted to minutes, and the money was converted to GBP. Time was also standardized for the last 7 days, which was done to match some of the periods of time the self-report measures focused on.

All other measures were standardized by subtracting the sample mean and dividing by the sample standard deviation for each measure, as specified in our pre-registration.

#### 2.3.2. Hypotheses 1–4

Vulnerable individuals are more likely to experience [H1: financial/H2: emotional/H3: vocational/educational/H4: sleep-related] outcomes of playing mobile games designed to drive spending, moderated by financial and time investment.

A single vulnerability score for all relevant measures (self-esteem, impulsiveness, general psychopathology, life satisfaction and job satisfaction) was computed. Self-esteem, life satisfaction and job satisfaction were reverse-scored for this calculation.

We had originally pre-registered and planned to run a multiple regression for hypotheses 1–4. Prior to these analyses, we checked the data for relevant assumptions: linearity, lack of outliers, homoscedasticity, normality of residuals, lack of multicollinearity and independence. However, for the majority of the measures, linearity and normality of residuals were not met. As detailed in the pre-registration, we next applied a series of transformations to the data, beginning with a log transformation (log base 10 of the data with an added constant of 1, to account for values which were zeroes). This was not successful in solving the above issues, and after some thought and visual inspection we decided a linear model would not be the most appropriate way to explain the data in this instance.

Therefore, in deviation from our pre-registration plan, we used a quantile regression, which can be used as a non-parametric extension of linear regression which does not require conditional assumptions regarding underlying distributions, to be used when the assumptions of linear regression are not met. It allows an understanding of the relationships outside of the mean of the data and does not have to follow the idea that variables behave the same at tails of the distribution as they do in the centre [36]. This regression is treated as exploratory, as it deviated from our original analysis plan.

#### 2.3.3. Hypotheses 5–6

[H5: Women/H6: People without children] are more likely than other genders to experience lower wellbeing outcomes of playing mobile games designed to drive spending, with time or financial investment as a covariate.

A single combined wellbeing outcome score was computed as the sum of scores for the measures of financial, emotional, physical (sleep) and education/employment wellbeing measures.

Similar issues regarding violation of statistical assumptions were faced with hypotheses 5–6, which were originally planned to be analysed with an ANCOVA. In this instance, the assumption of the independence of the covariate (time and money invested into game) and treatment effect (gender; presence of children) was not met. This meant we were unable to proceed with the model as planned, and—in deviation from our pre-registration plan—opted to remove the covariate, as is commonly recommended in such situations [37]. We thus ended up running an ANOVA rather than an ANCOVA, which is also treated as exploratory.

Prior to running an ANOVA, the three core assumptions of this test were also checked. Normality was checked through a Shapiro–Wilk test of normality and by plotting a histogram of the response variable; equality of variances was checked via boxplots. Independence of observations had been met by default, given the nature of our data collection. All of the assumptions were met and we were able to proceed with the ANOVA.

#### 2.3.4. Hypothesis 7

Players of games characterized as having had their dynamics designed to drive spending will be more likely to experience problematic gaming-related outcomes than players of other games.

This hypothesis was answered through a *t*-test, between the comparison group of players of other games and a randomly selected sample of the same size from the main body of participants, to ensure they were matched on other characteristics. The data were tested for normality using the Shapiro–Wilk test of normality, which was met.

## 3. Results

### 3.1. Descriptive statistics

For each measured variable, we present the sample mean, maximum and minimum, seen in table 1.

**Table 1.** The mean, maximum, minimum and standard deviation for each variable.

variable	mean	maximum	minimum	standard deviation
life satisfaction	14.31	31	1	7.22
self-esteem	3.84	7	1	1.67
psychopathology	37.38	110	0	25.72
impulsivity	62.8	107	34	10.80
job satisfaction	12.48	20	4	3.72
time spent playing over the last 7 days (minutes)	328.49	3844.4	0	560.45
money spent over the last 7 days (£)	0.72	35.084	0	3.78
financial wellbeing	36.83	68	10	9.59
job performance	39.81	51	24	5.00
sleep	5.19	10	1	2.25
emotional wellbeing	44.88	70	14	10.32

**Figure 2.**
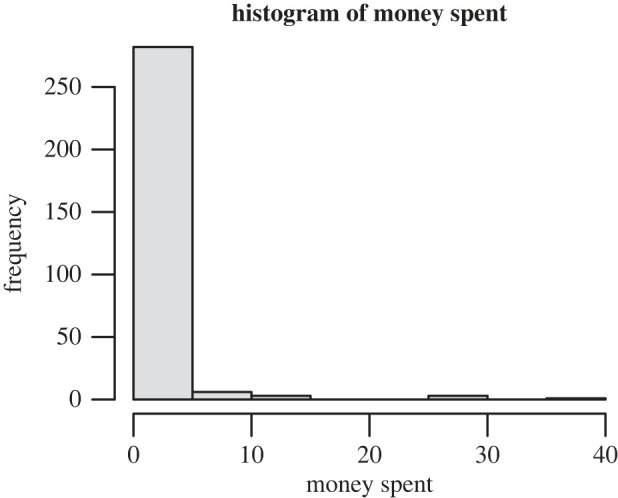
The distribution of money spent in our sample.

Another feature of the data worth highlighting at this stage is the extreme skewness and high prevalence of zeroes in the ‘money spent’ variable: only 22 of our 295 participants spent any money during the time period in question (7.5%) (figure 2).

While this does not affect our choice of analysis methods, given the lack of assumptions in regression about the distribution of the predictor variables, it should be borne in mind as the reader moves on to the results and discussion, as it reflects an underlying sample characteristic.

### 3.2. Hypotheses 1–4

A separate quantile regression was fitted for each of the hypotheses 1–4. We estimated the 0.1, the 0.25, the 0.75 the 0.9 quantile and the median. These choices were made to gain as comprehensive an understanding as possible of the relationships at different levels of the sample. The full results are presented in table 2.

**Table 2.** The coefficients and *p*-values for all predictors across all hypotheses at different tau levels. The * denotes significance at an alpha level of *p* < 0.01. The ** denotes significance at the Bonferroni-corrected level of *p <* 0.002 for multiple comparisons.

hypothesis	quantile (tau)	predictor	coefficient	*p*-value
1: financial wellbeing	0.1	vulnerability	0.05374	0.10269
		time	−0.00006	0.76979
		money	−0.07532	0.43293
		time*vulnerability	−0.00006	0.16405
		money*vulnerability	−0.01688	0.24112
	0.25	vulnerability	0.07634	0.00776*
		time	−0.00013	0.57448
		money	−0.01921	0.42006
		time*vulnerability	−0.00006	0.39041
		money*vulnerability	−0.00466	0.64629
	0.5	vulnerability	0.10487	0.00003**
		time	−0.00003	0.80335
		money	−0.02996	0.25071
		time*vulnerability	−0.00002	0.51453
		money*vulnerability	−0.00245	0.82208
	0.75	vulnerability	0.08544	0.00055*
		time	−0.00004	0.69257
		money	−0.02171	0.55991
		time*vulnerability	0.00000	0.97127
		money*vulnerability	0.00418	0.72661
	0.9	vulnerability	0.02843	0.30654
		time	−0.00018	0.10076
		money	0.00837	0.84350
		time*vulnerability	0.00001	0.73560
		money*vulnerability	0.01315	0.32949
2: emotional wellbeing	0.1	vulnerability	−0.19999	0.00000**
		time	0.00006	0.53081
		money	0.02481	0.04652
		time*vulnerability	0.00001	0.70803
		money*vulnerability	0.00906	0.05633
	0.25	vulnerability	−0.20692	0.00000**
		time	−0.00002	0.85593
		money	0.01963	0.18331
		time*vulnerability	0.00000	0.93167
		money*vulnerability	0.01119	0.04702
	0.5	vulnerability	−0.20765	0.00000**
		time	−0.00018	0.15952
		money	0.01041	0.55470
		time*vulnerability	0.00000	0.84759
		money*vulnerability	0.01385	0.04020
	0.75	vulnerability	−0.18221	0.00000**
		time	−0.00019	0.05716
		money	−0.00523	0.75497
		time*vulnerability	0.00000	0.94035
		money*vulnerability	0.00118	0.91705
	0.9	vulnerability	−0.19436	0.00000**
		time	−0.00002	0.93325
		money	0.02751	0.52523
		time*vulnerability	−0.00003	0.69356
		money*vulnerability	−0.00998	0.60570
3: educational/vocational wellbeing	0.1	vulnerability	−0.08965	0.00108*
		time	−0.00004	0.78743
		money	0.01408	0.61295
		time*vulnerability	−0.00004	0.38284
		money*vulnerability	0.00350	0.67695
	0.25	vulnerability	−0.08505	0.00197*
		time	0.00016	0.28694
		money	0.00364	0.86515
		time*vulnerability	−0.00001	0.69831
		money*vulnerability	0.00772	0.48714
	0.5	vulnerability	−0.07705	0.00972*
		time	0.00008	0.64064
		money	−0.00795	0.78339
		time*vulnerability	−0.00003	0.41045
		money*vulnerability	0.01298	0.37002
	0.75	vulnerability	−0.10640	0.00007**
		time	−0.00013	0.32474
		money	0.03457	0.57398
		time*vulnerability	0.00000	0.90457
		money*vulnerability	0.01465	0.39021
	0.9	vulnerability	−0.07002	0.00679**
		time	−0.00006	0.64955
		money	0.04111	0.53367
		time*vulnerability	−0.00002	0.55419
		money*vulnerability	0.02107	0.27136
4: sleep wellbeing	0.1	vulnerability	−0.08173	0.00064**
		time	−0.00008	0.50199
		money	0.00714	0.58499
		time*vulnerability	0.00002	0.31882
		money*vulnerability	0.00228	0.71088
	0.25	vulnerability	−0.09702	0.00110**
		time	−0.00018	0.27357
		money	−0.00168	0.92540
		time*vulnerability	0.00003	0.44910
		money*vulnerability	0.00604	0.46636
	0.5	vulnerability	−0.13927	0.00000**
		time	−0.00041	0.01135
		money	−0.02651	0.21777
		time*vulnerability	0.00003	0.37856
		money*vulnerability	−0.00063	0.94936
	0.75	vulnerability	−0.06819	0.02834
		time	−0.00040	0.00616*
		money	−0.05074	0.01217
		time*vulnerability	−0.00003	0.35566
		money*vulnerability	−0.00311	0.77316
	0.9	vulnerability	−0.07568	0.00331**
		time	−0.00029	0.01790
		money	−0.06455	0.00004**
		time*vulnerability	0.00003	0.42375
		money*vulnerability	−0.00301	0.71449

Table 2 illustrates the significance of all the variables across the above-described quantiles. Four models were fitted, one per hypothesis, and are denoted by the wellbeing outcome in question. By looking at the spread of quantiles in each model, the reader can consider whether the magnitude of the predictors and the interactions changes at all as the outcome variables move from the lower to the upper quantile.

We were interested in the significance and effect sizes of the interactions, namely, Time*Vulnerability and Money*Vulnerability, as those were the variables of interest in the hypotheses. Should these interactions have been significant, the hypothesis that ‘vulnerable’ individuals were more likely to experience wellbeing outcomes through moderation by greater playtime or greater in-game spend would have been met.

However, none of the interactions were significant at the pre-registered alpha level of *p* < 0.01, and even less so at the Bonferroni corrected level of *p* < 0.002 for multiple comparisons, which was implemented as an additional precaution given the amount of analyses being run. Therefore, we cannot conclude that ‘vulnerable’ individuals are more likely to experience financial, emotional, educational/vocational or sleep-related outcomes of playing mobile games designed to drive spending, moderated by financial and/or time investment. Nor were there any changes in significance across the quantiles, which suggests there is no difference even at the extreme ends of the outcome distributions.

The variable ‘vulnerability’ is consistently significant, but this is not relevant to our analyses, as it shows merely a link between certain psycho-environmental characteristics and wellbeing, which is already widely documented.

Because we standardized our variables prior to analysis, the coefficients of the predictors in quantile regression can be interpreted as expected change in the predictor when the response variable changes by 1 standard deviation. Our effect size of interest was 0.4 standard deviations, which was based on a paper by Norman *et al*. [38], who argue that ‘the threshold of discrimination for changes in health-related quality of life is half a standard deviation’ We adapted this effect size with the rationale that our study is about wellbeing and corrected it to 0.4 using Lord and Novick's [39] correction. None of the predictors showed an effect size of this magnitude. Moreover, none of the effect sizes could be categorized as meeting Cohen's *d* criteria for a small effect size of 0.2.

### 3.3. Hypotheses 5–6

A one-way ANOVA was performed to compare the effect of gender on the combined wellbeing outcomes. There was no statistically significant difference in wellbeing outcomes between females and other genders, *F*_1, 293_ = 0.015, *p* = 0.903. Another one-way ANOVA was fitted to compare the effect of having children on wellbeing outcomes, and was also not significant: *F*_1, 293_ = 3.522, *p* = 0.0616.

### 3.4. Hypothesis 7

Hypothesis 7 was tested by a *t*-test comparison of a random sample of players of ‘games designed to drive spending’ with players of other (non-mobile) games. The *t*-test was not significant: *t*_141.52_ = −1.0745, *p* = 0.2844, thus not concluding that players of games characterized as having had their dynamics designed to drive spending will be more likely to experience problematic gaming-related outcomes than players of other games.

### 3.5. Exploratory analyses

There was no significant difference between wellbeing outcomes in the players in the sample of interest who had invested the greatest amount of time playing (*n* = 72), and the least amount (*n* = 72): *t*_139.71_ = −2.3141, *p* = 0.02212.

There are no notable correlations between any of the ‘vulnerability’ variables and time or money spent, nor with wellbeing outcomes—with one exception: sleep wellbeing and playtime, at *r*_293_ = −0.18, *p* = 0.001.

## 4. Discussion

The work presented in this paper aimed to investigate the extent to which psycho-environmental characteristics are related to time, financial investment and wellbeing outcomes in a sample of players of games previously identified as ‘designed to drive spending’. We hypothesized that several characteristics would be important in this context: life satisfaction, self-esteem, job satisfaction, general psychopathology, impulsivity, gender and whether or not an individual has children they regularly see. Notably, we used objective evidence of time and financial investment into games—screenshot evidence provided by participants—rather than self-report of these measures.

However, in a sample of 295 players of ‘games designed to drive spending’, none of our hypotheses were met. There were no significant effects on any wellbeing-related outcomes as a result of any interaction between psycho-environmental characteristics and time/financial investment into games. This is a surprising finding, given that the study was based on a prior, qualitative piece of work which found the presence of financial, emotional, educational/vocational and sleep-related issues in a sample of individuals who demonstrated the psycho-environmental characteristics that inspired the present work. We must therefore explore several possible reasons for this discrepancy.

Firstly, we might conclude that psycho-environmental characteristics genuinely do not matter in this context, while not excluding the possibility that games designed to drive spending can cause harm to players—as our prior work suggests. To paraphrase, this simply means that an individual with higher levels of these characteristics is not more likely than someone with lower levels to spend more time playing or on microtransactions in the game. It is unlikely that we did not accurately capture at least a subset of the relevant characteristics that may have contributed to a relationship if there was one in this study, given we drew not only from our prior work, but from studies on inter- and intra-personal characteristics in gaming disorder [14] and in gambling [18]. Given the robustness of our study design, this conclusion seems likely, and we may hypothesize that the characteristics of those experiencing harms in our qualitative study could have been incidental.

If unwilling to conclude this, there are some possible other explanations for the divergence in findings. Potentially the most important of these relates to sampling. It may be the case that our recruitment strategy was not tuned to capture people for whom the relationship between psycho-environmental characteristics and wellbeing outcomes does hold. As mentioned above, our characteristics of interest are also commonly seen in studies in problem gambling and problem gaming. The key word here is ‘problem’: these associations are seen in small proportions of the population who show dysregulated behaviour. Meanwhile, our primary sample of interest was the normative gamer, and we were interested in answering the question of whether a normative individual can start playing a ‘game designed to drive spending’ and begin experiencing harm. It is possible that this work is a step forward in concluding that perhaps the ‘average’ individual will not experience harm if playing such a game. However, that does not mean that *nobody* will, and we must move onto studying outliers: players at extreme ends of the population in terms of psycho-environmental characteristics, or those who have a confirmed diagnosis of Internet gaming disorder, to understand whether game mechanics contributed to this dysregulation.

Moreover, one can argue that we may have got such findings because of the low proportion of participants in our sample who had actually spent any money in the game: after all, only 22 of our 295 participants had spent any money at all on games during the past 10 days, and only eight of these had spent more than £10. This is hardly representative of a group whose financial outlays are leading to important impacts on financial wellbeing. Our findings relating to spending as a moderator may be different if we surveyed only players who had spent, or only those who were engaged in heavy and sustained spending. In further work we will build on this by adding in recruitment criteria that participants must have spent in the game in a recent period of time—perhaps individuals who feel inclined to spend on ‘games designed to drive spending’ do show different relationships. Nonetheless, this does not render our findings invalid. There is probably generally a very low rate of individuals who do regularly spend in such games [40], and as such our study of the normative player holds. Notedly, this lack of variability in engagement was not the case when it came to playtime data: while spending data were heavily zero-inflated, we had a normally distributed sample of players regarding how much time they had spent playing, meaning we captured some players who had invested a lot of time. This is also an important factor.

A final potential explanation of the divergence between this study and prior results concerns the timing of these pieces of research. The participants in the prior study discussed their experiences over a certain period of time, and for many of them, this period included the peak of the COVID-19 pandemic. It is universally accepted that the pandemic was disruptive to people's ordinary lives and mental health, not least because of the lockdowns imposed in the majority of countries. This also led to changes in how and why people played games, for example, increased quantity of play for multi-player games [41]. People were also drawn to games such as *Animal Crossing: New Horizons* for temporary escapism and social connection [42]. Therefore, perhaps people played mobile games designed to drive spending more and for different reasons in the height of the pandemic, and were also experiencing higher levels of ‘vulnerability’ because of the environmental pressures around them. This may not have been captured by the sample in the current study, surveyed at what is a calmer time (in many English-speaking countries).

This brings us onto an analytical limitation of the current study, in that it is cross-sectional. This means we were able to capture only a snapshot of our participants' behaviours, feelings and experiences at a current moment in time, whereas behaviours such as dysregulated gaming and technology-related wellbeing outcomes play out over a much longer period. In the future, we would like to conduct longitudinal research on this issue, to understand whether there is a link between time and financial investment into games, psycho-environmental characteristics and wellbeing which develops over time in players of ‘games designed to drive spending’.

Another analytical constraint is that the effect size of interest, 0.4, was originally calculated based on 314 participants, whereas our sample contains 295 (the reason for the change is resource-based and is outlined in the pre-registration document). Moreover, perhaps a medium-sized effect was too great an assumption: if small effects existed, the current sample may not have picked up on them. The rationale behind using this effect size was based on a paper by Norman *et al*. [38] conceptualization of half a standard deviation being a robust effect size for health-related research. However, we accept we may have been over-eager in our desire to align conceptualizations of wellbeing in this study with those described by Norman *et al*., and our future work is considering effect sizes more thoroughly (see https://osf.io/85tyc/).

Interestingly, we also did not find a significant difference in the level of wellbeing outcomes experienced by players of ‘games designed to drive spending’ and players of other, non-mobile games. This finding should be treated less conclusively, as the control sample of ‘other’ games did not undergo an additional categorization process. Furthermore, the control sample consisted entirely of non-mobile games: this was based on rationale from previous work [6] that non-mobile games are less likely to be ‘designed to drive spending’, but does pose a limitation in that we did not control for differing play styles or other confounds in non-mobile games. It, therefore, warrants further investigation in our future work (see pre-registration above). However, it may be an initial indication once again that the normative player will not experience harm from interacting with game mechanics designed to drive their spending more so than from playing other games. This is a reassuring finding but still points to a need to study players at the more extreme ends of the spectrum.

## 5. Conclusion

Games which have had their ‘dynamics designed to drive spending’ are a worrying and prevalent phenomenon. The current study investigated whether certain types of players are more likely to invest more time and money into such games, and as a result more likely to experience negative wellbeing across several life areas. We found no evidence to suggest this, nor that there is a significant difference between players of such games and alternative games in their wellbeing.

This is a hopeful finding, as it may suggest that the majority of players are not affected by the design elements employed to encourage their spending and excessive play. However, it is at odds with our previous—qualitative—work which suggested a link was present. Several reasons may underlie this discrepancy, such as the link being present only in more extreme populations and a need for longitudinal research. We plan to address this in our future work.

## Data Availability

Readers can access a synthetic dataset for the main sample, the control sample and the code used for analysis at https://osf.io/ma56y/?view_only=e1e55d6e1cd146e6967825f505f92054 (made using the ‘synthpop’ R package). The datasets were synthesized prior to being standardized, and the synthesized variables were then standardized and summed. We reran our analyses on the synthetic datasets, and found very similar results. This, coupled with visual inspection of the variable distribution of the synthetic variables alongside the original, leaves us confident the synthetic datasets closely resemble the original data. Supplementary material is available online [43].

## References

[1] Zanescu A, French M, Lajeunesse M. 2021 Betting on DOTA 2's Battle Pass: gamblification and productivity in play. New Media Soc. **23**, 2882-2901. (10.1177/1461444820941381)

[2] Whitson J, French M. 2021 Productive play: the shift from responsible consumption to responsible production. J. Consum. Cult. **21**, 14-33. (10.1177/1469540521993922)

[3] Delfabbro P, King DL. 2020 Gaming-gambling convergence: evaluating evidence for the ‘gateway' hypothesis. Int. Gambl. Stud. **20**, 380-392. (10.1080/14459795.2020.1768430)

[4] Brock T, Johnson M. 2021 The gamblification of digital games. J. Consum. Cult. **21**, 3-13. (10.1177/1469540521993904)

[5] Petrovskaya E, Zendle D. 2021 Predatory monetisation? A categorisation of unfair, misleading, and aggressive monetisation techniques in digital games from the perspective of players. J. Bus. Ethics **181**, 1065-1081. (10.1007/s10551-021-04970-6)

[6] Petrovskaya E, Deterding CS, Zendle D. 2021 Prevalence and salience of problematic microtransactions in top-grossing mobile and PC games: a content analysis of user review. In *Proc. of the 2022 CHI Conf. on Human Factors in Computing Systems (CHI '22), New Orleans, LA, 29 April–5 May*. New York, NY: Association for Computing Machinery.

[7] Zendle D. 2020 Beyond loot boxes: a variety of gambling-like practices in video games are linked to both problem gambling and disordered gaming. PeerJ **8**, e9466. (10.7717/peerj.9466) 32742782 PMC7367055

[8] Petrovskaya E, Zendle D. 2022 ‘These people had taken advantage of me’: a grounded theory of problematic consequences of player interaction with mobile games perceived as ‘designed to drive spending’. Hum. Behav. Emerg. Technol. **2022**, 1260174. (10.1155/2022/1260174)

[9] Vuorre M, Johannes N, Magnusson K, Przybylski AK. 2022 Time spent playing video games is unlikely to impact well-being. R. Soc. Open Sci. **9**, 220411. (10.1098/rsos.220411) 35911206 PMC9326284

[10] Carras MC, Van Rooij AJ, Van de Mheen D, Musci R, Xue QL, Mendelson T. 2017 Video gaming in a hyperconnected world: a cross-sectional study of heavy gaming, problematic gaming symptoms, and online socializing in adolescents. Comput. Hum. Behav. **68**, 472-479. (10.1016/j.chb.2016.11.060) PMC5330315 28260834

[11] Kristensen JH, Pallesen S, King DL, Hysing M, Erevik EK. 2021 Problematic gaming and sleep: a systematic review and meta-analysis. Front. Psychiatry **12**, 675237. (10.3389/fpsyt.2021.675237) 34163386 PMC8216490

[12] Mihara S, Higuchi S. 2017 Cross-sectional and longitudinal epidemiological studies of Internet gaming disorder: a systematic review of the literature. Psychiatry Clin. Neurosci. **71**, 425-444. (10.1111/pcn.12532) 28436212

[13] Krossbakken E, Pallesen S, Mentzoni RA, King DL, Molde H, Finserås TR, Torsheim T. 2018 A cross-lagged study of developmental trajectories of video game engagement, addiction, and mental health. Front. Psychol. **9**, 2239. (10.3389/fpsyg.2018.02239) 30519203 PMC6258776

[14] Bargeron AH, Hormes JM. 2017 Psychosocial correlates of internet gaming disorder: psychopathology, life satisfaction, and impulsivity. Comput. Hum. Behav. **68**, 388-394. (10.1016/j.chb.2016.11.029)

[15] Cardoso J, Ferreira T, Dores A. 2021 The psychological determinants of internet gaming disorder: vulnerability to stress, psychological well-being, and comorbidity. Eur. Psychiatry. **64**, S172-S173. (10.1192/j.eurpsy.2021.458)

[16] Bean AM, Nielsen RK, Van Rooij AJ, Ferguson CJ. 2017 Video game addiction: the push to pathologize video games. Prof. Psychol. Res. Pract. **48**, 378. (10.1037/pro0000150)

[17] Schüll ND. 2012 Addiction by design: machine gambling in Las Vegas. Princeton, NJ: Princeton University Press.

[18] Sharman S, Butler K, Roberts A. 2019 Psychosocial risk factors in disordered gambling: a descriptive systematic overview of vulnerable populations. Addict. Behav. **99**, 106071. (10.1016/j.addbeh.2019.106071) 31473572

[19] Abbott M. 2020 The changing epidemiology of gambling disorder and gambling-related harm: public health implications. Public Health **184**, 41-45. (10.1016/j.puhe.2020.04.003) 32402593

[20] Kahn AS, Ratan R, Williams D. 2014 Why we distort in self-report: predictors of self-report errors in video game play. J. Comput.-Mediat. Commun. **19**, 1010-1023. (10.1111/jcc4.12056)

[21] Hampton KN. 2017 Studying the digital: directions and challenges for digital methods. Annu. Rev. Sociol. **43**, 167-188. (10.1146/annurev-soc-060116-053505)

[22] Ohme J, Araujo T, de Vreese CH, Piotrowski JT. 2021 Mobile data donations: assessing self-report accuracy and sample biases with the iOS screen time function. Mob. Media Commun. **9**, 293-313. (10.1177/2050157920959106)

[23] Wardle H, Zendle D. 2021 Loot boxes, gambling, and problem gambling among young people: results from a cross-sectional online survey. Cyberpsychol. Behav. Soc. Netw. **24**, 267-274. (10.1089/cyber.2020.0299) 33103911 PMC8064953

[24] Ioannidis K, Hook R, Wickham K, Grant JE, Chamberlain SR. 2019 Impulsivity in gambling disorder and problem gambling: a meta-analysis. Neuropsychopharmacology **44**, 1354-1361. (10.1038/s41386-019-0393-9) 30986818 PMC6588525

[25] Nowok B, Raab GM. 2016 Dibben C. synthpop: bespoke creation of synthetic data in R. J. Stat. Softw. **74**, 1-26. (10.18637/jss.v074.i11)

[26] Quintana DS. 2020 A synthetic dataset primer for the biobehavioural sciences to promote reproducibility and hypothesis generation. Elife **9**, e53275, (10.7554/eLife.53275) 32159513 PMC7112950

[27] Diener E, Emmons RA, Larsen RJ, Griffin S. 1985 The satisfaction with life scale. J. Pers. Assess. **49**, 71-75. (10.1207/s15327752jpa4901_13) 16367493

[28] Robins RW, Hendin HM, Trzesniewski KH. 2001 Measuring global self-esteem: construct validation of a single-item measure and the Rosenberg Self-Esteem Scale. Pers. Soc. Psychol. Bull. **27**, 151-161. (10.1177/0146167201272002)

[29] Patton JH, Stanford MS, Barratt ES. 1995 Factor structure of the Barratt impulsiveness scale. J. Clin. Psychol. **51**, 768-774. (10.1002/1097-4679(199511)51:6<768::AID-JCLP2270510607>3.0.CO;2-1) 8778124

[30] Antony MM, Bieling PJ, Cox BJ, Enns MW, Swinson RP. 1998 Psychometric properties of the 42-item and 21-item versions of the depression anxiety stress scales in clinical groups and a community sample. Psychol. Assess. **10**, 176. (10.1037/1040-3590.10.2.176)

[31] Thompson ER, Phua FT. 2012 A brief index of affective job satisfaction. Group Organ. Manag. **37**, 275-307. (10.1177/1059601111434201)

[32] Prawitz A, Garman ET, Sorhaindo B, O'Neill B, Kim J, Drentea P. 2006 InCharge financial distress/financial well-being scale: development, administration, and score interpretation. J. Financ. Couns. Plan. **17**, 34-50.

[33] Stewart-Brown S, Janmohamed K. 2008 *Warwick-Edinburgh mental well-being scale:* *user guide version 1*. See http://www.mentalhealthpromotion.net/resources/user-guide.pdf.

[34] Carlos VS, Rodrigues RG. 2016 Development and validation of a self-reported measure of job performance. Soc. Indic. Res. **126**, 279-307. (10.1007/s11205-015-0883-z)

[35] Snyder E, Cai B, DeMuro C, Morrison MF, Ball W. 2018 A new single-item sleep quality scale: results of psychometric evaluation in patients with chronic primary insomnia and depression. J. Clin. Sleep Med. **14**, 1849-1857. (10.5664/jcsm.7478) 30373688 PMC6223557

[36] Hao L, Naiman DQ, Naiman DQ. 2007 Quantile regression. London, UK: Sage.

[37] Miller GA, Chapman JP. 2001 Misunderstanding analysis of covariance. J. Abnorm. Psychol. **110**, 40. (10.1037/0021-843X.110.1.40) 11261398

[38] Norman GR, Sloan JA, Wyrwich KW. 2003 Interpretation of changes in health-related quality of life: the remarkable universality of half a standard deviation. Med. Care **41**, 582-592. (10.1097/01.mlr.0000062554.74615.4c) 12719681

[39] Lord FM, Novick MR. 2008 Statistical theories of mental test scores. Charlotte, NC: IAP.

[40] Close J, Spicer SG, Nicklin LL, Uther M, Lloyd J, Lloyd H. 2021 Secondary analysis of loot box data: are high-spending ‘whales’ wealthy gamers or problem gamblers? Addict. Behav. **117**, 106851. (10.1016/j.addbeh.2021.106851) 33578105

[41] Vuorre M, Zendle D, Petrovskaya E, Ballou N, Przybylski AK. 2021 A large-scale study of changes to the quantity, quality, and distribution of video game play during a global health pandemic. Technol. Mind Behav. **2**. (10.1037/tmb0000048)

[42] Zhu L. 2021 The psychology behind video games during COVID-19 pandemic: a case study of *Animal Crossing: New Horizons*. Hum. Behav. Emerg. Technol. **3**, 157-159. (10.1002/hbe2.221)

[43] Petrovskaya E, Zendle D. 2023 The relationship between psycho-environmental characteristics and wellbeing in non-spending players of certain mobile games. Figshare. (10.6084/m9.figshare.c.6387877) PMC9874280 36704251

